# Discovery of VU6008677: A Structurally Distinct Tricyclic M_4_ Positive Allosteric Modulator with Improved CYP450 Profile

**DOI:** 10.1021/acsmedchemlett.4c00249

**Published:** 2024-07-03

**Authors:** Rory A. Capstick, Sean R. Bollinger, Julie L. Engers, Madeline F. Long, Sichen Chang, Vincent B. Luscombe, Alice L. Rodriguez, Colleen M. Niswender, Thomas M. Bridges, Olivier Boutaud, P. Jeffrey Conn, Darren W. Engers, Craig W. Lindsley, Kayla J. Temple

**Affiliations:** † Warren Center for Neuroscience Drug Discovery, Vanderbilt University, Nashville, Tennessee 37232, United States; ‡ Department of Pharmacology, Vanderbilt University School of Medicine, Nashville, Tennessee 37232, United States; § Department of Chemistry, Vanderbilt University, Nashville, Tennessee 37232, United States; ∥ Vanderbilt Kennedy Center, Vanderbilt University School of Medicine, Nashville, Tennessee 37232, United States; ⊥ Vanderbilt Brain Institute, Vanderbilt University School of Medicine, Nashville, Tennessee 37232, United States

**Keywords:** M_4_, Muscarinic Acetylcholine Receptor Subtype 4, Positive Allosteric Modulator (PAM), Structure−Activity Relationship (SAR)

## Abstract

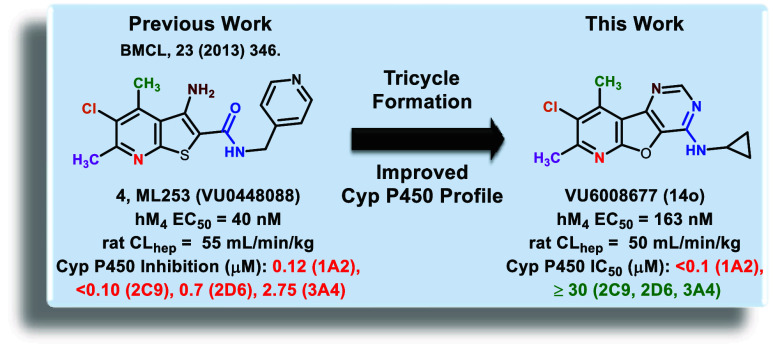

This Letter details our efforts to develop novel tricyclic muscarinic acetylcholine receptor subtype 4 (M_4_) positive allosteric modulator (PAM) scaffolds with improved pharmacological properties. This endeavor involved a “tie-back” strategy to replace the 3-amino-5-chloro-4,6-dimethylthieno[2,3-*b*]pyridine-2-carboxamide core, which led to the discovery of two novel tricyclic cores: an 8-chloro-9-methylpyrido[3′,2′:4,5]thieno[3,2-*d*]pyrimidin-4-amine core and 8-chloro-7,9-dimethylpyrido[3′,2′:4,5]furo[3,2-*d*]pyrimidin-4-amine core. Both tricyclic cores displayed low nanomolar potency against human M_4_ and greatly reduced cytochrome P450 inhibition when compared with parent compound **ML253**.

Muscarinic acetylcholine receptor subtype 4 (M_4_) positive allosteric modulators (PAMs) continue to be important drug targets as novel treatments for various neurological disorders, such as Parkinson’s disease,^1^ Huntington’s disease,^2^ and schizophrenia (both the positive and negative symptom clusters).^3−7^ Traditional M_4_ PAMs possess a β-amino carboxamide moiety, which was believed to be a key pharmacophore required for M_4_ PAM activity (Figure 1, dashed circle).^8−17^ This particular chemotype engendered poor solubility in earlier M_4_ PAMs, varying degrees of P-gp efflux, and potency discrepancies across species. Therefore, there has been much focus on the development of novel M_4_ PAM chemotypes devoid of the β-amino carboxamide moiety.^18−30^ For instance, xanomeline, an M_1_/M_4_-preferring agonist lacking the β-amino carboxamide moiety, has been evaluated in clinical trials. These trials have given further validation to targeting the muscarinic cholinergic system as a treatment for the psychosis and behavioral disturbances observed in both Alzheimer’s and schizophrenia patients.^25,26^ Xanomeline’s lack of selectivity among receptor subtypes resulted in adverse events and ultimately the discontinuation of clinical development. An effort to overcome these side effects led to the development of KarXT, which was recently submitted as a New Drug Application to the FDA. KarXT is a treatment in which trospium (a pan-selective peripheral muscarinic acetylcholine receptor antagonist) is coadministered with xanomeline to counteract the adverse events of xanomeline administration alone.^27^ Recently, a selective M_4_ PAM was shown to not only be efficacious in preclinical assays but also exhibited fewer and less severe adverse cholinergic-related side effects when compared with rats treated with the nonselective M_4_ agonist xanomeline.^28^ These data suggest that receptor-subtype-selective M_4_ PAMs exhibit improved safety profiles compared with agonists, and CVL-231 (a selective M_4_ PAM) is currently in clinical testing.^29,30^

**Figure 1:**
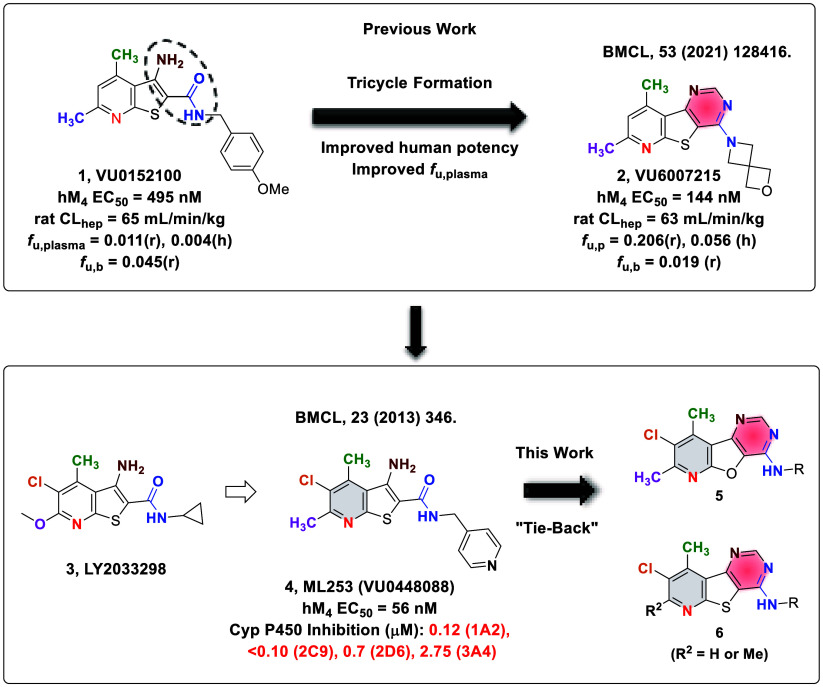
Exploration of novel tricyclic cores as M_4_ PAMs revealed two unique M_4_ PAM tricyclic chemotypes: 8-chloro-7,9-dimethylpyrido[3′,2′:4,5]furo[3,2-*d*]pyrimidin-4-amine (**5**) and 8-chloro-9-methylpyrido[3′,2′:4,5]thieno[3,2-*d*]pyrimidin-4-amine (**6**).

Our lab has focused on developing novel M_4_ PAM chemotypes devoid of the β-amino carboxamide moiety, several of which were recently disclosed.^14−16^ Most recently, we described a novel 6,5,6-tricyclic scaffold that still afforded potent and CNS-penetrant M_4_ PAMs (Figure 1, **VU6007215**). To identify additional novel tricyclic M_4_ PAM chemotypes, we elected to further explore the “tie-back” strategy, which aids in masking the detrimental β-amino carboxamide moiety (an essential pharmacophore in earlier M_4_ PAMs). Using an historical M_4_ PAM (**ML253**) developed by our laboratory as a starting point, we employed this “tie-back” strategy to generate novel tricyclic cores.^16,31^ This resulted in the discovery of a novel M_4_ PAM chemotype containing an 8-chloro-7,9-dimethylpyrido[3′,2′:4,5]furo[3,2-*d*]pyrimidin-4-amine core, **5**. Further exploration revealed a second novel, tricyclic M_4_ PAM chemotype containing an 8-chloro-9-methylpyrido[3′,2′:4,5]thieno[3,2-*d*]pyrimidin-4-amine core, **6**. This body of work details the development of these two novel M_4_ PAM chemotypes.

The synthesis of tricyclic cores **5** and **6** began with a Gewald-type reaction (Scheme 1). Treatment of ethyl 2-mercaptoacetate or ethyl 2-hydroxyacetate with commercially available nicotinonitriles **7a** or **7b** under basic conditions with irradiation in a microwave reactor afforded carboxylates **10a**–**c**. Treatment of a heated solution of intermediates **10a**–**c** in formamide with a formamidine acetate salt gave pyrimidone intermediates **11a**–**c**. Pyrimidones **11a**–**c** were then converted into chlorides **12a**–**c** with POCl_3_, which then readily underwent nucleophilic aromatic substitutions with a variety of amines to yield desired analogues **13**–**15**. For this work, we decided to forego exploring amino azetidines of past M_4_ PAMs because of their potential metabolic instability.^15^ Instead, we focused on examining small aliphatic amines, as well as benzyl amines, that could aid in increasing solubility. When indicated, HCl salts of final analogues were prepared to aid in improving solubility.

**Scheme 1:**
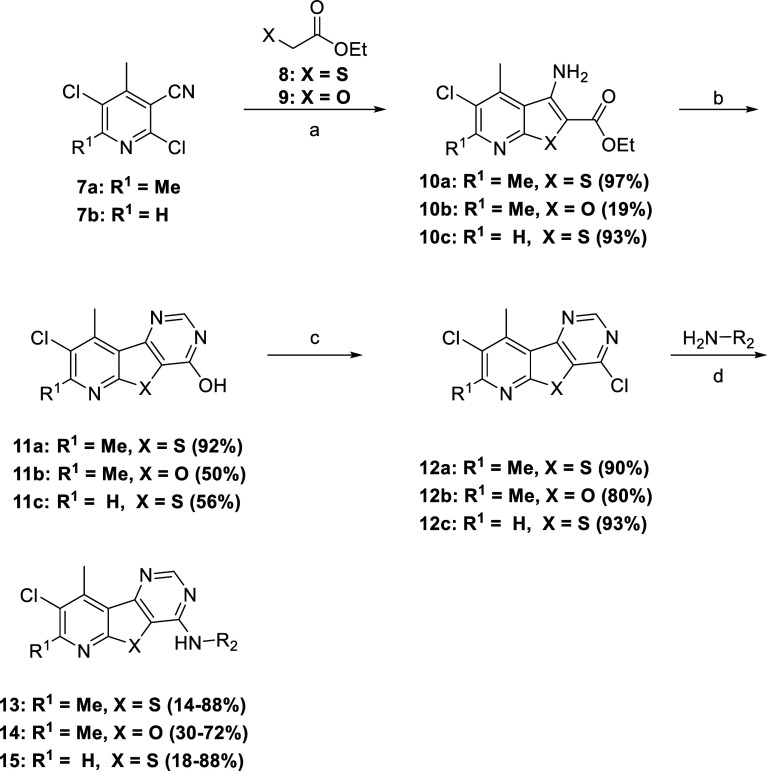
Synthesis of M_4_ PAM analogues **13–15** Reagents and conditions: (a) K_2_CO_3_ or Cs_2_CO_3_, isopropyl alcohol (IPA) or *N*-methyl-2-pyrrolidone (NMP), microwave irradiated at, 150 °C; (b) formamidine acetate salt, formamide, NMP, 150 °C; (c) POCl_3_, 120 °C; (d) (i) amine, *N*,*N*-diisopropylethylamine (DIEA), NMP, 50 °C; (ii) HCl, DCM/MeOH (5:1).

We next turned our attention to evaluating the relevance of the pyrimidine nitrogen at the 5-position of core **6** by synthesizing analogues **22** (Scheme 2). To synthesize tricyclic core **6**, intermediate **10a** underwent a copper-catalyzed Sandmeyer reaction to yield bromide **16**, which could then undergo Suzuki–Miyaura coupling with 1,3,2-dioxaborolane **17** to afford carboxylate **18**. Intermediate **18** then underwent ozonolysis to give aldehyde **19**, which was subjected to hydrazine to afford pyridazinone **20**. Treatment with POCl_3_ yielded chloride **21**, which could readily undergo nucleophilic aromatic substitution to generate desired analogues **22**.

**Scheme 2:**
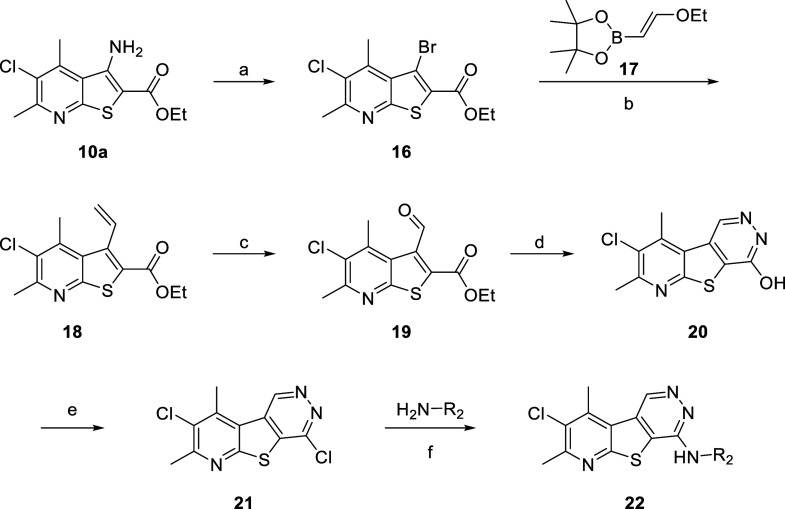
Synthesis of M_4_ PAM Analogues **22** Reagents and conditions: (a) CuBr_2_, ^*t*^BuONO, MeCN, 88%; (b) Pd(dppf)Cl_2_, Cs_2_CO_3_, 1,4-dioxane/H_2_O (10:1), 90 °C, 64%; (c) (i) O_3_, DCM, −78 °C; (ii) dimethyl sulfide (DMS), 38%; (d) H_2_NNH_2_, EtOH/IPA (1:1), 110 °C, 86%; (e) POCl_3_, microwave irradiated at 150 °C, 74%; (f) (i) amine, DIEA, NMP, 50 °C, 19–79%; (ii) HCl, DCM/MeOH (5:1).

Initially, select analogues **13** were screened against human M_4_ (hM_4_) to determine potency with results highlighted in Table 1. These results highlight the importance of the amine tail on the potency. In the context of the 8-chloro-7,9-dimethylpyrido[3′,2′:4,5]thieno[3,2-*d*]pyrimidine core (**5**), the benzylamine-containing analogues **13a**–**c** all have a hM_4_ EC_50_ > 2.5 μM or were inactive. The presence of hydrogen bond acceptors in the “benzyl-like” tails (**13a** and **13b**) were detrimental to activity. Interestingly, the analogue with a larger amine tail, **13h** (hM_4_ EC_50_ = 1.5 μM), was more potent than the benzylamine analogues. This perhaps indicates that not only size but orientation of the tail within the allosteric pocket plays a key role in potency. Several of the small aliphatic amine tails provided compounds with a hM_4_ EC_50_ < 750 nM (**13j**, hM_4_ EC_50_ = 740 nM; **13k**, hM_4_ EC_50_ = 740 nM; and **13l**, hM_4_ EC_50_ = 72 nM). It was observed that minor modifications, such as methyl substitutions on the cyclopropyl amine (**13g** vs **13l** and **13k** vs **13l**), led to 26-fold and 10-fold losses in potency, respectively. Moreover, reducing the size of the amine tail by replacing the 2-oxa-6-azaspiro[3.3]heptane ring of **13d** (hM_4_ EC_50_ > 10 μM) with pyrrolidine (**13e**, hM_4_ EC_50_ = 5.9 μM) resulted in a modest increase in potency. This trend was observed as the amine tail was further reduced in size to azetidine (**13f**, hM_4_ EC_50_ = 3.1 μM) and ultimately the cyclopropyl amine (**13l**, hM_4_ EC_50_ = 72 nM).

**Table 1:**
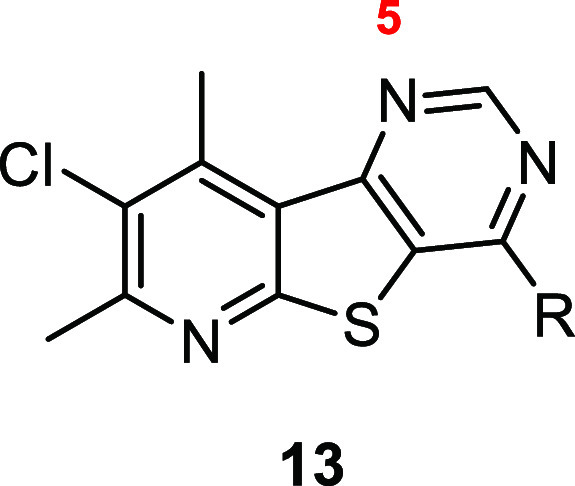

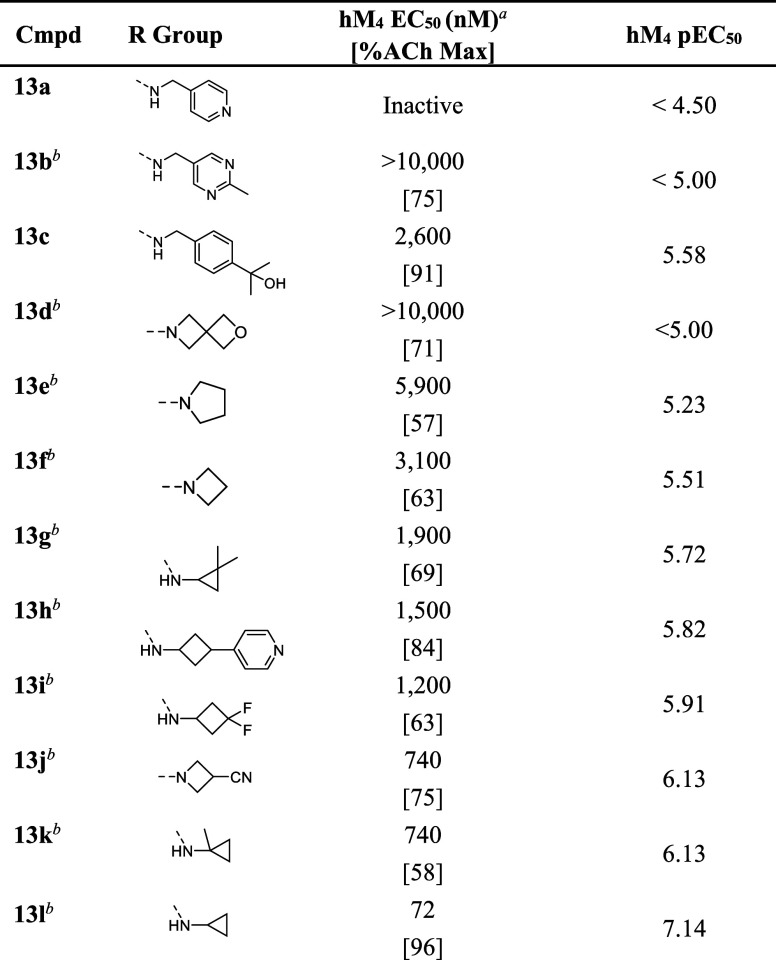
Structures and Activities for Analogues **13**

a Calcium mobilization assays with hM_4/Gqi5_-CHO cells were performed in the presence of a EC_20_ fixed concentration of acetylcholine. Half-maximal effective concentration (EC_50_) values for hM_4_ represents at least one experiment performed in triplicate.

b The HCl salt form was utilized.

Next, we turned our attention toward accessing the importance of sulfur in the 8-chloro-7,9-dimethylpyrido [3′,2′:4,5]thieno[3,2-*d*]pyrimidine core (**6**). To do so, we exchanged the sulfur with an oxygen to generate an 8-chloro-7,9-dimethylpyrido[3′,2′:4,5]furo[3,2-*d*]pyrimidine core (**5**, analogues **14**). Select analogues were screened against hM_4_ to determine potency, with results highlighted in Table 2. In general, analogues with core **14** provided more potent compounds compared with analogues **13**. Most notably, (2-methylpyrimidin-5-yl)methanamine analogues (**14f** vs **13b**), pyrrolidine analogues (**14b** vs **13e**), azetidine analogues (**14l** vs **13f**), 3,3-difluorocyclobutan-1-amine analogues (**14j** vs **13i**), and 2,2-dimethylcyclopropan-1-amine analogues (**14p** vs **13g**) led to a >8-fold, 2.4-fold, 8.6-fold, 3-fold, and 17-fold increase in potency, respectively.

**Table 2:**
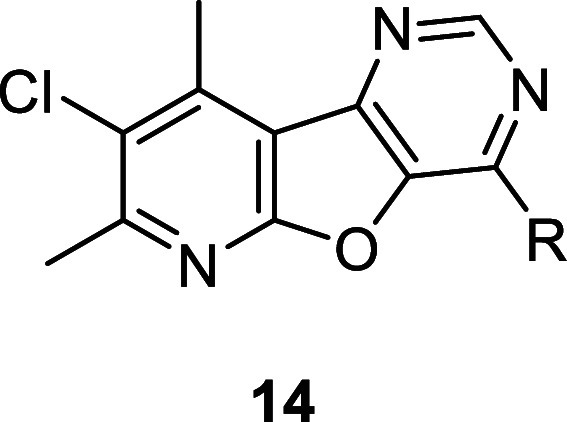

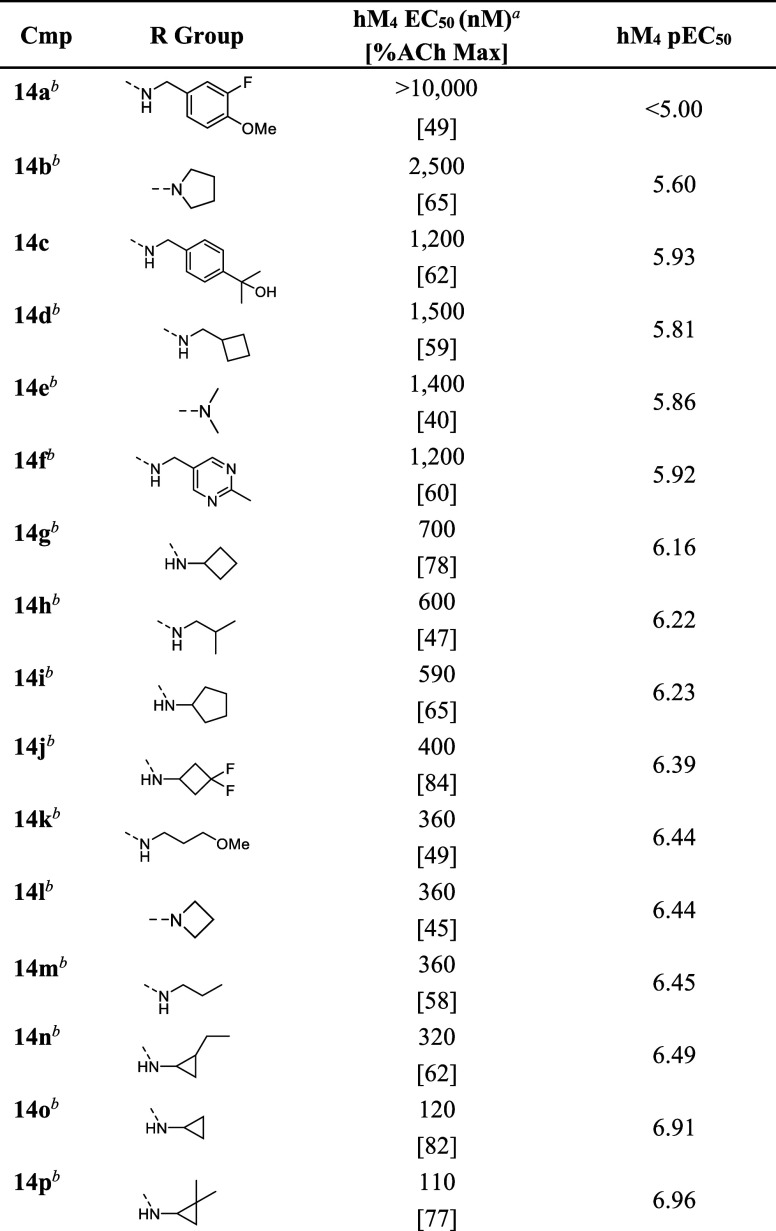
Structures and Activities for Analogues **14**

a Calcium mobilization assays with hM_4/Gqi5_-CHO cells were performed in the presence of an EC_20_ fixed concentration of acetylcholine. EC_50_ values for hM_4_ represent at least one experiment performed in triplicate.

b The HCl salt form was utilized.

Subsequently, we investigated the relevance of the methyl substituent ortho to the pyridine of core **6**. Toward this end, select analogues **15** were screened against hM_4_ to determine potency with results highlighted in Table 3. Interestingly, removal of the methyl at the 2-position of the tricycle gave rise to several analogues with a hM_4_ EC_50_ ≤ 450 nM. Most notably, several amine tails that are inactive or have a hM_4_ EC_50_ > 10 μM in the context of core **13** (analogues **13a, b, d**) gave rise to potent compounds (**15c**, hM_4_ EC_50_ = 450 nM; **15d**, hM_4_ EC_50_ = 320 nM; **15h**, hM_4_ EC_50_ = 29 nM).

**Table 3:**
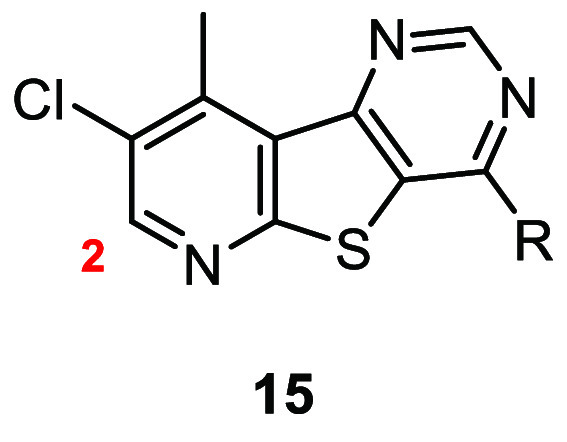

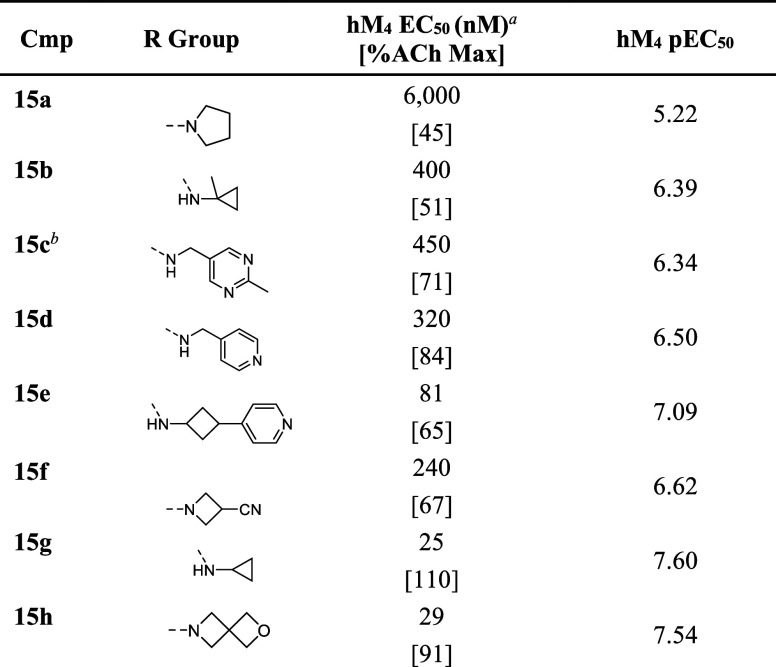
Structures and Activities for Analogues **15**

a Calcium mobilization assays with hM_4/Gqi5_-CHO cells were performed in the presence of an EC_20_ fixed concentration of acetylcholine. EC_50_ values for hM_4_ represent at least one experiment performed in triplicate.

b The HCl salt form was utilized.

Finally, we investigated the significance of nitrogen placement within the pyrimidine ring of core **6**. Select analogues **22** were screened in tandem against hM_4_ to determine potency with results highlighted in Table 4. Similarly, small aliphatic amine tails provided the most potent analogues, with a hM_4_ EC_50_ ≤ 200 nM (**22h**, hM_4_ EC_50_ = 150 nM; **22i**, hM_4_ EC_50_ = 65 nM; **22j**, hM_4_ EC_50_ = 83 nM; and **22k**, hM_4_ EC_50_ = 39 nM). It was noted that minor changes, such as a fluoro-substitution on the cyclobutanamine tail (**22k** vs **22j**), led to a 2-fold increase in potency. Like analogues **13**, reducing the size of the amine tail by replacing the piperidine ring of **22b** (hM_4_ EC_50_ = 860 nM) with pyrrolidine (**22d**, hM_4_ EC_50_ = 430 nM) resulted in a modest increase in potency. This trend was observed as the amine tail was further reduced in size to azetidine (**22h**, hM_4_ EC_50_ = 150 nM) and the cyclopropyl amine (**22i**, hM_4_ EC_50_ = 65 nM). As there is a 2-fold difference in hM_4_ potency between **22h** and **22i**, it can be inferred that a hydrogen bond donor is not required for activity at this position. The addition of a methylene spacer to analogue **22j** (hM_4_ EC_50_ = 83 nM) resulted in a nearly 5-fold decrease in potency (**22f**, hM_4_ EC_50_ = 400 nM). Apart from the cyclopropanamine analogues (**13l** and **22i**), moving the nitrogen from the 5-position of the tricycle to the 6-position generally resulted in more potent analogues. For instance, comparing the pyrrolidine analogues (**13e vs 22d**), the azetidine analogues (**13f** vs **22h**), and the 3,3-difluorocyclobutan-1-amine analogues (**13i** vs **22k**) resulted in a 14-fold, 21-fold, and 31-fold increase in hM_4_ potency, respectively.

**Table 4:**
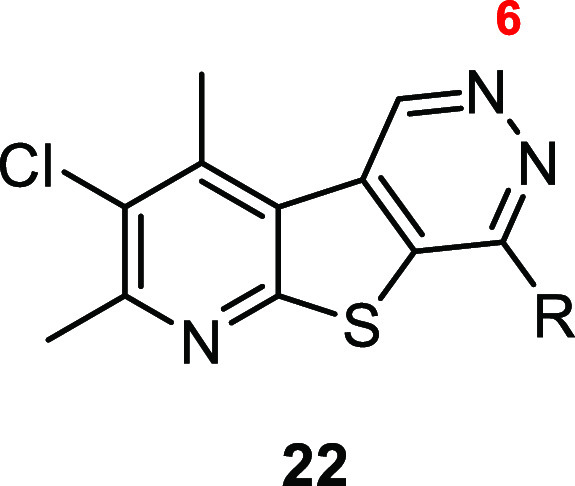

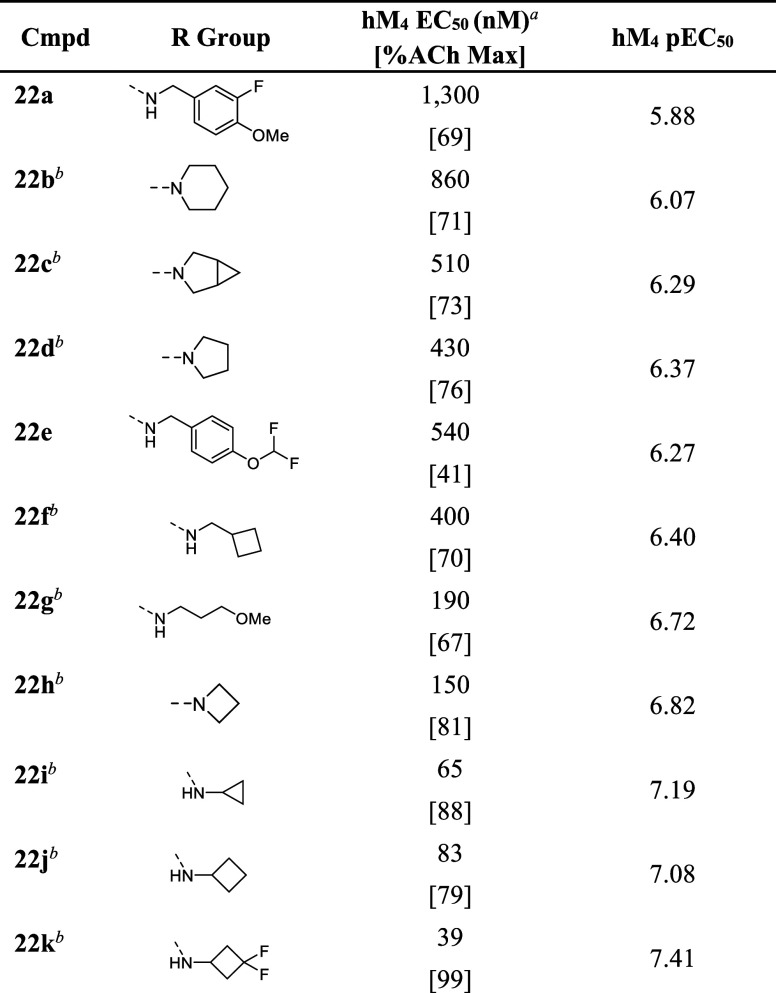
Structures and Activities for Analogues **22**

a Calcium mobilization assays with hM_4/Gqi5_-CHO cells were performed in the presence of an EC_20_ fixed concentration of acetylcholine. EC_50_ values for hM_4_ represent at least one experiment performed in triplicate.

b The HCl salt form was utilized.

Of these compounds, **13l**, **14o**–**p**, **15e**–**h**, and **22i**–**k** were advanced into a battery of *in vitro* drug metabolism and pharmacokinetics (DMPK) assays (Table 5). In regards to physiochemical properties, these analogues all possessed molecular weights less than 450 Da, as well as several analogues having attractive CNS xLogP values (2.79–3.39).^32,33^ All compounds tested displayed high predicted hepatic clearance (CL_hep_) in rat microsomes (rat CL_hep_*s* > 47 mL/min/kg). While analogue **14o** displayed moderate human predicted hepatic clearance on the basis of microsomal data (human CL_hep_ 13 mL/min/kg), all other analogues tested displayed high human-predicted hepatic clearance (human CL_hep_ ≥ 15 mL/min/kg).

**Table 5:** *In vitro* DMPK Data for Select Analogues **13, 14, 15**, and **22**

property	**13l**	**14o**	**14p**	**15e**	**15f**	**15g**	**15h**	**22i**	**22j**	**22k**
	**VU6008810**	**VU6008677**	**VU6009105**	**VU6008462**	**VU6008455**	**VU6056615**	**VU6008460**	**VU6008650**	**VU6008346**	**VU6008649**
MW	304.8	288.73	316.79	367.86	315.78	290.77	332.81	304.8	318.82	354.81
xLogP	3.63	3.06	3.48	3.29	2.6	3.43	2.27	3.06	3.51	3.73
topological polar surface area (TPSA)	50.7	64.1	64.1	54.8	65.7	50.37	51.1	50.7	50.7	50.7
hM_2_ EC_50_ (μM) [% ACh Max]^a^	5.0 [49]	inactive	2.3 [39]	inactive	inactive	0.48 [40]	inactive	inactive	>10 [44]	0.26 [49]
***in vitro* pharmacokinetics parameters**										
CL_int_ (mL/min/kg), rat	423	175	467	316	642	789	334	420	1275	1674
CL_hep_ (mL/min/kg), rat	60	50	61	57	63	64	58	60	66	67
CL_int_(mL/min/kg), human	78	37	168	79	224	57	147	52	49	185
CL_hep_ (mL/min/kg), human	17	13	19	17	19	15	18	15	15	19
rat *f*_u*,*plasma_^b^	0.010	0.060	0.038	0.010	0.171	0.039	0.031	0.042	0.026	0.057
human *f*_u*,*plasma_^b^	0.004	0.007	0.004	0.010	0.020	0.010	0.008	0.013	0.005	0.010
rat *f*_u*,*brain_^b^	0.001	0.017	0.002	0.005	0.002	0.003	0.003	0.011	0.006	0.008
CYP450 (μM)^c^										
1A2	<0.1	<0.1	0.32	1.5	0.36	<0.1	0.64	<0.1	0.52	0.98
2C9	30	>30	>30	9.1	>30	>30	>30	19.2	>30	>30
2D6	>30	>30	>30	0.33	>30	>30	>30	7.9	19.2	25.2
3A4	15.8	>30.0	>30	>30	>30	24.3	>30	28.8	>30	>30

a Calcium mobilization assays with hM_2/Gqi5_-CHO cells were performed in the presence of an EC_20_ fixed concentration of acetylcholine. EC_50_ values for hM_2_ represent one or two independent experiments performed in triplicate. hM_2_ = human M_2_

b *f*_u_ = fraction unbound; equilibrium dialysis assay; brain = rat brain homogenates;

c Assayed in pooled human liver microsomes (HLM) in the presence of NADPH with CYP-specific probe substrates.

As indicated by the fraction unbound (*f*_u_), compounds **13l** and **15e** were highly bound to plasma proteins and rat brain homogenate (rat and human *f*_u,plasma_ ≤ 0.010; rat *f*_u,__brain_ = 0.001–0.005). By contrast, **14p**, **15g**, **15h**, **22i**, and **22j** were moderately bound to rat plasma proteins (rat *f*_u,plasma_ = 0.026–0.042); however, all were highly bound to human plasma proteins (human *f*_u,plasma_ = 0.004–0.012). Additionally, all were highly bound to rat brain homogenate (rat *f*_u,brain_ = 0.002–0.011). Interestingly, compounds **14o** (rat *f*_u,plasma_ = 0.060; rat *f*_u,brain_ = 0.017; human *f*_u,plasma_ = 0.007) and **15f** (rat *f*_u,plasma_ = 0.171; rat *f*_u,brain_ = 0.002; human *f*_u,plasma_ = 0.020) displayed the best protein binding profiles; however, **15f** displayed high human and rat predicted hepatic clearance (human CL_hep_ = 19 mL/min/kg; rat CL_hep_ = 63 mL/min/kg). By contrast, analogue **14o** displayed moderate human predicted hepatic clearance (CL_hep_ = 13 mL/min/kg) and high rat predicted hepatic clearance (rat CL_hep_ = 50 mL/min/kg). Most notably, the tricyclic analogues **13**–**15** generally exhibit an improved cytochrome (CYP) P450 inhibition profile when compared with **ML253** (**4**, Figure 1). In fact, in some instances (**13l**, **14o**, **15f**–**h**, and **22k**), we observed a drastic improvement regarding CYPs 2C9, 2D6, and 3A4 inhibition (IC_50_ values > 15.8 μM). Unfortunately, **14o** offered no improvement to CYP 1A2 inhibition (IC_50_ < 0.1 μM), while **15f** provided only a modest 3-fold improvement of CYP 1A2 inhibition (IC_50_ = 0.36 μM) when compared with **ML253**. Moreover, both **14o** and **15h** were inactive when screened on human M_2_.

In summary, a scaffold hopping exercise utilizing a “tie-back” strategy based on M_4_ PAM **4** proved to be a successful strategy in converting an early lead compound **ML253** into potent, tricyclic M_4_ PAM analogues devoid of the classic β-amino carboxamide moiety. Unlike an earlier observation noted with the **VU6007215** tricyclic scaffolds, a thiophene as the central ring was not greatly favored over a furan in the newer tricycle series (**13l** vs **14o**).^22^ To achieve highly potent compounds within these tricyclic series (**13**, **14**, **15**, and **22**), aliphatic amines were preferred over benzyl amines. Unfortunately, many of these potent analogues displayed very poor rat brain and human plasma protein fraction unbound (*f*_u_ values < 0.01), as well as high human (CL_hep_ values ≥ 15 mL/min/kg) and rat (CL_hep_ values ≥ 46 mL/min/kg) predicted hepatic clearance. Interestingly, masking the β-amino carboxamide moiety as a tricycle was an effective strategy to drastically improve the CYP450 inhibition profile in comparison with lead **ML253**.

Overall, **14o** (**VU6008677**) displayed the best pharmacokinetics (PK) profile with moderate human predicted hepatic clearance (CL_hep_ = 13 mL/min/kg), moderate rat brain homogenate binding (*f*_u,brain_ = 0.017), favorable rat plasma protein binding (*f*_u,plasma_ = 0.060), and selectivity over hM_2_. Compound **14o** also provided an improved CYP inhibition profile (CYP 2C9, 2D6, and 3A4 IC_50_ values ≥ 30 μM) when compared with parent compound **ML253**. However, **VU6008677** was not progressed forward because of high predicted rat microsomal clearance, high human plasma protein binding (*f*_u,plasma_ < 0.01), and CYP 1A2 inhibition (IC_50_ < 0.10 μM). Although this exercise did not provide M_4_ PAMs with suitable DMPK profiles to warrant further advancement, it did highlight SAR insights for future scaffold designs. These refinements will be reported in due course.

## Supporting Information Available

General methods for the synthesis and characterization for key compounds and experimental details for calcium mobilization assays and *in vivo* DMPK protocols (PDF)

## Abbreviations

ACh: acetylcholine
CL_hep_: hepatic clearance
CL_int_: intrinsic clearance
CNS: central nervous system
CYP: cytochrome P450
DMPK: drug metabolism and pharmacokinetics
FDA: Food & Drug Administration
hM_4_: muscarinic acetylcholine receptor subtype 4, human
PAM: positive allosteric modulator
P-gp: P-glycoprotein
SAR: structure–activity relationship
