# Supplementary material for: Discovery of VU6008677: A Structurally Distinct Tricyclic M4 Positive Allosteric Modulator with Improved CYP450 Profile
Source: ACS Med Chem Lett. 2024 Jul 3;15(8):1358–66. doi: 10.1021/acsmedchemlett.4c00249 (PMC11318023; doi:10.1021/acsmedchemlett.4c00249)
Supplement: Supplementary file 1 — ml4c00249_si_001.pdf [file ml4c00249_si_001.pdf]

Supplemental Information for

Discovery of VU6008677: A Structurally Distinct Tricyclic M_4 Positive Allosteric Modulator with Improved CYP450 Profile.

Rory A. Capstick[a,b], Sean R. Bollinger[a,b], Julie L. Engers[a,b], Madeline F. Long[a,b], Sichen Chang[a,b], Vincent B. Luscombe[a,b], Alice L. Rodriguez[a,b], Colleen M. Niswender[a,b,d,e], Thomas M. Bridges[a,b], Olivier Boutaud[a,b], P. Jeffrey Conn[a,b,d], Darren W. Engers[a,b], Craig W. Lindsley[a,b,c], Kayla J. Temple[a,b*]

[a]Warren Center for Neuroscience Drug Discovery, Vanderbilt University, Nashville, TN 37232, USA
[b]Department of Pharmacology, Vanderbilt University School of Medicine, Nashville, TN 37232, USA
[c]Department of Chemistry, Vanderbilt University, Nashville, TN 37232, USA
[d]Vanderbilt Kennedy Center, Vanderbilt University School of Medicine, Nashville, TN 37232, USA
[e]Vanderbilt Brain Institute, Vanderbilt University School of Medicine, Nashville, TN 37232, USA

Corresponding Author Information:

[*]Corresponding author: Kayla J. Temple – Department of Pharmacology, Vanderbilt University, Nashville, TN 37232, USA. ORCID: 0000-0001-5290-574X. Email: kayla.temple@vanderbilt.edu

Table of Contents

Experimental Synthetic Procedures and Spectroscopic Data S2
General Synthetic Methods S2
General Instrumentation Methods. S2
General Procedure for the Preparation of Analogs **13** (Scheme 1). S3
General Procedure for the Preparation of Analogs **14** (Scheme 1). S5
General Procedure for the Preparation of Analogs **22** (Scheme 2). S8
Molecular Pharmacology Methods S10
Calcium Mobilization Assays. S10
DMPK Methods S12
Binding in plasma from rat and human. S12
Binding in brain homogenate from rat S12
Intrinsic Clearance in Rat and Human Liver Microsomes S13
LC-MS/MS Analysis S14
References S15

Experimental Synthetic Procedures and Spectroscopic Data

General Synthetic Methods.

All reactions were carried out employing standard chemical techniques. Solvents used for extraction, washing, and chromatography were HPLC grade. All reagents were purchased from commercial sources and were used without further purification.

Automated flash column chromatography was performed on a Biotage Isolera 1 or a Teledyne ISCO CombiFlash system. RP-HPLC was performed on a Gilson preparative reversed-phase HPLC system comprised of a 333 aqueous pump with solvent-selection valve, 334 organic pump, GX-271 or GX-281 liquid hander, two column switching valves, and a 155 UV detector. Absorbance was typically monitored at 215 or 220 nm. Column: Phenomenex Axia-packed Gemini C18, 5 µm. Mobile phase: CH_3CN in H_2O (0.1% TFA) or CH_3CN in H_2O (0.05% *v/v* NH_4OH) under the specified gradient, then hold 95% CH_3CN in 5% aqueous phase, 50 mL/min, 23° C. All compounds were found to be >95% pure by LCMS analysis.

***Safety statement**: no unexpected or unusually high safety hazards were encountered.*

General Instrumentation Methods.

All NMR spectra were recorded on a 400 MHz AMX Bruker NMR spectrometer. ^1H and ^{13}C chemical shifts are reported in δ values in ppm downfield with the deuterated solvent as the internal standard. Data are reported as follows: chemical shift, multiplicity (s = singlet, d = doublet, t = triplet, q = quartet, b = broad, m = multiplet), integration, coupling constant (Hz).

Low resolution mass spectra (LRMS) were obtained on an Agilent 6120/6150 or Waters QDa (Performance) SQ MS with ESI source. *Method A (Agilent 6120/6150):* MS parameters were as follows: fragmentor: 70, capillary voltage: 3000 V, nebulizer pressure: 30 psig, drying gas flow: 13 L/min, drying gas temperature: 350 ºC. Samples were introduced via an Agilent 1290 UHPLC comprised of a G4220A binary pump, G4226A ALS, G1316C TCC, and G4212A DAD with ULD flow cell. UV absorption was generally observed at 215 nm and 254 nm with a 4 nm bandwidth. Column: Waters Acquity BEH C18, 1.0 x 50 mm, 1.7 um. Gradient conditions: 5% to 95% CH_3CN in H_2O (0.1% TFA) over 1.4 min, hold at 95% CH_3CN for 0.1 min, 0.5 mL/min, 55 ºC. *Method B (Agilent 6120/6150):* MS parameters were as follows:

fragmentor: 100, capillary voltage: 3000 V, nebulizer pressure: 40 psig, drying gas flow: 11 L/min, drying gas temperature: 350 ºC. Samples were introduced via an Agilent 1200 HPLC comprised of a degasser, G1312A binary pump, G1367B HP-ALS, G1316A TCC, G1315D DAD, and a Varian 380 ELSD (if applicable). UV absorption was generally observed at 215 nm and 254 nm with a 4 nm bandwidth. Column: Thermo Accucore C18, 2.1 x 30 mm, 2.6 um. Gradient conditions: 7% to 95% CH_3CN in H_2O (0.1% TFA) over 1.6 min, hold at 95% CH_3CN for 0.35 min, 1.5 mL/min, 45 ºC. *Method C (Waters QDa (Performance) SQ MS)*: MS parameters were as follows: cone voltage: 15 V, capillary voltage: 0.8 kV, probe temperature: 600° C. Samples were introduced via an Acquity I-Class PLUS UPLC comprised of a BSM, FL-SM, CH-A, and PDA. UV absorption was generally observed at 215 nm and 254 nm; 4 nm bandwidth. Column: Phenomenex EVO C18, 1.0 x 50 mm, 1.7 um. Column temperature: 55° C. Flow rate: 0.4 mL/min. Default gradient: 5% to 95% CH_3CN (0.05% TFA) in H_2O (0.05% TFA) over 1.4 min (curve 6), hold at 95% CH_3CN for 0.1 min. "Polar" (2% to 70% CH_3CN (0.05% TFA) in H_2O (0.05% TFA) over 0.8 min (curve 6), transition to 95% CH_3CN over 0.1 min (curve 6), hold at 95% CH_3CN for 0.6 min.) and "Non-Polar" (40% to 95% CH_3CN (0.05% TFA) in H_2O (0.05% TFA) over 1.4 min (curve 6), hold at 95% CH_3CN for 0.1 min.) gradients were also available. *Method D (Waters QDa (Performance) SQ MS):* MS parameters were as follows: cone voltage: 15 V, capillary voltage: 0.8 kV, probe temperature: 600° C. Samples were introduced via an Acquity I-Class PLUS UPLC comprised of a BSM, FL-SM, CH-A, and PDA. UV absorption was generally observed at 215 nm and 254 nm with a 4 nm bandwidth. Column: Phenomenex EVO C18, 1.0 x 50 mm, 1.7 um. Column temperature: 55° C. Flow rate: 0.4 mL/min. Default gradient: 5% to 95% CH_3CN in H_2O (5 mM NH_4HCO_3) over 1.4 min (curve 6), hold at 95% CH_3CN for 0.1 min. "Polar" (2% to 70% CH_3CN in H_2O (5 mM NH_4HCO_3) over 0.8 min (curve 6), transition to 95% CH_3CN over 0.1 min (curve 6), hold at 95% CH_3CN for 0.6 min.) and "Non-Polar" (40% to 95% CH_3CN in H_2O (5 mM NH_4HCO_3) over 1.4 min (curve 6), hold at 95% CH_3CN for 0.1 min.) gradients were also available.

High resolution mass spectra (HRMS) were obtained on an Agilent 6540 UHD Q-TOF with ESI source. MS parameters were as follows: fragmentor: 150, capillary voltage: 3500 V, nebulizer pressure: 60 psig, drying gas flow: 13 L/min, drying gas temperature: 275 ºC. Samples were introduced via an Agilent 1200 UHPLC comprised of a G4220A binary pump, G4226A 3 ALS, G1316C TCC, and G4212A DAD with ULD flow cell. UV absorption was observed at 215 nm and 254 nm with a 4 nm bandwidth. Column: Agilent Zorbax Extend C18, 1.8 µm, 2.1 x 50 mm. Gradient conditions: 5% to 95% CH_3CN in H_2O (0.1% formic acid) over 1 min, hold at 95% CH_3CN for 0.1 min, 0.5 mL/min, 40 ºC.

<u>General Procedure for the Preparation of Analogs **13** (Scheme 1).</u>

*Synthesis of Intermediate **10a**:*

2,5-Dichloro-4,6-dimethylnicotinonitrile (**7a**) (2.00 g, 9.9 mmol, 1.0 eq.), ethyl thioglycolate (1.3 mL, 12 mmol, 1.2 eq.) and K_2CO_3 (2.80 g, 20 mmol, 2.0 eq.) were dissolved in IPA (10 mL) and the solution was microwave irradiated to 150 °C for 30 minutes. After cooling to ambient temperature, the reaction mixture was diluted with water and a precipitate was observed. The mixture was stirred for 20 minutes at room temperature and the solids collected by vacuum filtration washing with water (3x) to afford title compound (2.78 g, 91% yield). ^{1}H NMR (400 MHz, DMSO-d_6) δ 6.87 (s, 2H), 4.28 (q, J = 7.1 Hz, 2H), 2.83 (s, 3H), 2.63 (s, 3H), 1.29 (t, J = 7.0 Hz, 3H); LRMS: $C_{12}H_{13}ClN_2O_2S$ $[M+H]^+$ calc. mass 285.0, found 284.9.

*Synthesis of Intermediate **11a***:

A solution of intermediate **10a** (3.36 g, 0.12 mmol, 1.0 eq.) in formamide (45 mL) and NMP (15 mL) was heated to 150 °C. At 150 °C, formamidine acetate (24.6 g, 240 mmol, 20 eq.) was added to the reaction in 12 equal portions over 6 hours. The reaction was allowed to stir overnight at 150 °C then additional formamidine acetate (12.3 g, 120 mmol, 10 eq.) was added to the reaction in 6 equal portions over 3 hours. The reaction was removed from heat and cooled to ambient temperature and 200 mL of water was added while stirring. The mixture was allowed to sit overnight, and precipitate was collected by vacuum filtration. After washing with water, the solid was dried under nitrogen to provide the title compound (2.89 g, 92% yield) as a brown solid which was directly used in next step without purification. LRMS: $C_{11}H_8ClN_3OS$ $[M+H]^+$ calc. mass 265.0, found 266.2.

*Synthesis of Intermediate **12a**:*

Intermediate **11a** (2.89 g, 11 mmol) in $POCl_3$ (15 mL) was allowed to stir at 120 °C. After 5 hours, the solution was cooled to room temperature. The reaction mixture was slowly transferred in several portions to a large beaker containing saturated aqueous $NaHCO_3$ solution (100 mL) at 0 °C and stirred vigorously. The pH was routinely monitored and maintained at pH > 6 with the addition of solid K_2CO_3. After complete addition, the mixture was allowed to stir until gas evolution ceased. The solid precipitate was then collected by vacuum filtration washing with water and allowed to dry under nitrogen to provide the title compound (2.78 g, 90% yield) as a brown solid. ^{1}H NMR (400 MHz, DMSO-d_6) δ 9.21 (d, J = 1.3 Hz, 1H), 3.04 (d, J = 1.3 Hz, 3H), 2.73 (d, J = 1.3 Hz, 3H); LRMS: $C_{11}H_7Cl_2N_3S$ $[M+H]^+$ calc. mass 284.0, found 284.2.

*Synthesis of Compound **13l (VU6008810)**:*

To a suspension of cyclopropylamine (73.0 µL, 1.1 mmol, 2.0 eq.) in NMP (5 mL) was added *N*,*N*-diisopropylethylamine (368 µL, 2.1 mmol, 4.0 eq.) followed by intermediate **12a** (150 mg, 0.53 mmol, 1.0

eq.). The reaction mixture was stirred at 50 °C for 2 hours then cooled to room temperature. The reaction mixture was purified using RP-HPLC (70-100% ACN/water/0.1% TFA). Fractions containing pure product were basified with saturated aqueous $NaHCO_3$ solution and extracted with DCM (3x). The combined organics were passed through a phase separator and concentrated. The resulting solid was resuspended in 20 mL of DCM/MeOH (5:1) and 4*M* HCl in dioxane (5.0 mL) was added. The mixture was allowed to stir for 30 minutes then concentrated to afford title compound (147 mg, 74% yield). ^{1}H NMR (400 MHz, DMSO-d_6) δ 8.66 (s, 1H), 8.11 (d, J = 3.2 Hz, 1H), 3.09 (s, 3H), 3.07 – 2.96 (m, 1H), 2.70 (s, 3H), 0.88 – 0.75 (m, 2H), 0.71 – 0.63 (m, 2H). ^{13}C NMR (101 MHz, DMSO-d_6) δ 158.34, 158.05, 156.35, 154.64, 153.55, 143.85, 129.01, 124.60, 113.44, 23.98, 23.67, 15.53, 6.69 (2C); HRMS: $C_{14}H_{13}ClN_4S$ $[M+1]^+$ calc. mass 305.0622, found, 305.0621.

General Procedure for the Preparation of Analogs **14** (Scheme 1).

Synthesis of Intermediate ***10b****:*

2,5-Dichloro-4,6-dimethylnicotinonitrile (**7a**) (1.00 g, 6.0 mmol, 1.0 eq.), ethyl glycolate (0.60 mL, 6.6 mmol, 1.1 eq.) and Cs_2CO_3 (5.90 g, 18 mmol, 3.0 eq.) were dissolved in NMP (9.2 mL) and allowed to stir at 75 °C overnight. The reaction mixture was filtered through a pad of Celite® which was rinsed with DCM and EtOAc. The filtrate was concentrated and purified using RP-HPLC to provide title compound (250 mg, 16% yield). ^{1}H NMR (400 MHz, DMSO-d_6): δ 6.09 (s, 2H), 4.34 (q, J = 7.0 Hz, 2H), 2.72 (s, 3H), 2.61 (s, 3H), 1.34 (t, J = 7.0 Hz, 3H); LRMS: $C_{12}H_{13}ClN_2O_3$ $[M+H]^+$ calc. mass 269.1, found 269.2.

Synthesis of Intermediate ***11b****:*

A solution of intermediate **10b** (250 mg, 0.93 mmol, 1.0 eq.) in formamide (1.7 mL) was heated to 150 °C. At 150 °C, formamidine acetate (290 mg, 2.8 mmol, 3.0 eq.) was added to the reaction every 30 minutes for 1.5 hours, for a total of 9 equivalents. The reaction was removed from heat and cooled to ambient temperature. The solution was added to H_2O and was extracted with $CHCl_3$/IPA (3:1). The organic layers were combined and concentrated under reduced pressure to produce the title compound (230 mg, 99% yield) as a brown solid which was directly used in next step without purification. LRMS: $C_{11}H_8ClN_3O_2$ $[M+H]^+$ calc. mass 250.0, found 250.2.

Synthesis of Intermediate ***12b****:*

Intermediate **11b** (232 mg, 0.93 mmol) in $POCl_3$ (2.6 mL) was allowed to stir at 120 °C. After 4 hours, the solution was cooled to room temperature and transferred to a large beaker. Saturated aqueous $NaHCO_3$ solution (100 mL) was added followed by solid K_2CO_3 in increments, allowing the gas production to be

controlled while stirring vigorously until the pH ~ 8 was obtained. The reaction mixture was extracted with EtOAc (3x). The organic layers were combined, dried (Mg_2SO_4), filtered and concentrated under reduced pressure to provide the title compound (200 mg, 80 % yield) as a brown solid. ^{1}H NMR (400 MHz, DMSO–d_6): δ 9.14 (s, 1H), 2.98 (s, 3H), 2.77 (s, 3H); LRMS: $C_{11}H_7Cl_2N_3O$ $[M+H]^+$ calc. mass 268.0, found 268.2.

*Synthesis of compound **14o (VU6008677)** (Scheme 1, Table 3):*

To a solution of cyclopropylamine (75.0 mg, 1.3 mmol, 5.0 eq.) in NMP (1.8 mL) was added *N*,*N*-diisopropylethylamine (182 µL, 1.1 mmol, 4.0 eq.) followed by intermediate **12b** (70.1 mg, 0.26 mmol, 1.0 eq.). The reaction mixture was stirred at 50 °C for 2 hours then cooled to room temperature. The reaction mixture was purified using RP- HPLC (30-80% ACN/water/0.05% NH_4OH). Fractions containing pure product were concentrated and the resulting solid was resuspended in 2 mL of DCM/MeOH (5:1) and 4*M* HCl in dioxane (1.3 mL) was added. The mixture was allowed to stir for 5 minutes then concentrated to afford title compound (85 mg, 99% yield). ^{1}H NMR (400 MHz, CD_3OD) δ 9.07 (s, 1H), 3.61 – 3.48 (m, 1H), 3.31 (s, 3H), 3.15 (s, 3H), 1.51 – 1.37 (m, 2H), 1.30 – 1.20 (m, 2H). ^{13}C NMR (101 MHz, CD_3OD) δ 161.22, 160.16, 150.58, 148.09, 145.56, 140.01, 134.34, 130.36, 111.93, 25.26, 24.34, 17.13, 7.40; HRMS: $C_{14}H_{13}ClN_4O$ $[M+H]^+$ calc. mass 289.0851, found 289.0853.

*Synthesis of compound **14p (VU6009105)** (Scheme 1, Table 3):*

Prepared in a similar manner as analog **14o** to afford title compound as an off-white solid (5.9 mg, 45% yield). ^{1}H NMR (400 MHz, DMSO-d_6) δ 8.50 (s, 1H), 8.24 (s, 1H), 2.84 (s, 3H), 2.76 (s, 1H), 2.64 (s, 3H), 1.17 (s, 3H), 0.96 (s, 3H), 0.76 (dd, *J* = 7.9, 5.2 Hz, 1H), 0.69 (d, *J* = 4.7 Hz, 1H). ^{13}C NMR (101 MHz, DMSO-d_6) δ 159.27, 155.20, 153.86, 150.24, 150.13, 143.25, 133.60, 127.11, 112.60, 35.29, 24.63, 23.71, 19.04, 18.71, 17.78, 15.89; HRMS: $C_{16}H_{17}ClN_4O$ $[M+H]^+$ calc. mass 317.1164, found 317.1167.

General Procedure for the Preparation of Analogs **15** (Scheme 1).

*Synthesis of Intermediate **10c**:*

2,5-dichloro-4-methylnicotinonitrile (**7b**) (1.12 g, 6.0 mmol, 1.0 eq.), ethyl thioglycolate (790 µL, 7.2 mmol, 1.2 eq.) and K_2CO_3 (1.7 g, 12 mmol, 2.0 eq.) were dissolved in IPA (24mL) and microwave irradiated 105 °C for 30 minutes. After cooling to ambient temperature, the reaction mixture was diluted with water (~10 mL), stirred for 15 minutes, and allowed to settle for 2-3 hours. The precipitate was then collected by vacuum filtration and washed with water. The material was dried overnight under vacuum to afford title compound as a yellow powder which was directly used in the next step without further purification (1.52

g, 93% yield). ^{1}H NMR (400 MHz, DMSO-d_6) δ 8.63 (d, J = 2.6 Hz, 1H), 6.88 (s, 2H), 4.29 (q, J = 7.1 Hz, 2H), 2.83 (s, 3H), 1.30 (t, J = 7.1 Hz, 3H); LRMS: $C_{11}H_{11}ClN_2O_2S$ $[M+H]^+$ calc. mass 271.0, found 270.9.

Synthesis of Intermediate ***11c*** *(Scheme 1)*:

A solution of intermediate **10c** (1.52 g, 5.6 mmol, 1.0 eq.) in formamide (30 mL) was heated to 150 °C. At 150 °C, formamidine acetate (1.75 g, 17 mmol, 3.0 eq.) was added to the reaction every hour, for a total of 9 equivalents. The reaction was allowed to heat for 3 hours then additional formamidine acetate (1.75 g, 16.8 mmol, 3.0 eq.) was added and the reaction was stirred for 16 h. The reaction was then recharged twice more with formamidine acetate (1.75 g, 17 mmol, 3.0 eq.) over 6 hours. After cooling to room temperature, the reaction mixture was poured into H_2O and a precipitate was formed. The solid was collected by vacuum filtration, washing with water, and dried under vacuum overnight to provide title compound as a light gray powder which was directly used in next step without purification (1.2 g, 57% yield). ^{1}H NMR (400 MHz, DMSO-d_6) δ 8.77 (s, 1H), 8.42 (s, 1H), 3.04 (s, 3H), OH proton is not observable; LRMS: $C_{10}H_6ClN_3OS$ $[M+H]^+$ calc. mass 252.0, found 251.9.

Synthesis of Intermediate ***12c*** *(Scheme 1):*

Intermediate **11c** (251 mg, 0.66 mmol, 1.0 eq.) in $POCl_3$ (3.3 mL) was allowed to stir at 120 °C. After 2 hours, the solution was cooled to room temperature and diluted with DCM. Saturated aqueous $NaHCO_3$ solution was added until the pH ~8 was obtained. The mixture was extracted with DCM (3x). The organic layers were combined, dried (Mg_2SO_4), filtered and concentrated under reduced pressure to provide the title compound as a yellow solid which was used directly without further purification (200 mg, 80% yield). ^{1}H NMR (400 MHz, DMSO-d_6) δ 9.30 (s, 1H), 8.95 (s, 1H), 3.13 (s, 3H); LRMS: $C_{10}H_5Cl_2N_3S$ $[M+H]^+$ calc. mass 270.0, found 269.9.

Preparation of ***15f (VU6008455)*** *(Scheme 1, Table 4):*

To a suspension of azetidine-3-carbonitrile hydrochloride (59.0 mg, 0.50 mmol, 5.0 eq.) in NMP (1.0 mL) was added *N*,*N*-diisopropylethylamine (122 μL, 0.70 mmol, 7.0 eq.) followed by intermediate **12c** (13.0 mg, 0.10 mmol, 1.0 eq.). The reaction mixture was stirred at 50 °C for 2-3 hours then cooled to room temperature. The reaction mixture was purified using RP-HPLC to afford title compound (18.5 mg, 59% yield) as an off-white solid. ^{1}H NMR (400 MHz, DMSO-d_6) δ 8.77 (s, 1H), 8.68 (s, 1H), 4.68 (dd, J = 8.8, 8.8 Hz, 2H), 4.57 (dd, J = 8.5, 6.0 Hz, 2H), 4.04 (tt, J = 9.0, 6.0 Hz, 1H), 3.06 (s, 3H). ^{13}C NMR (101 MHz, DMSO-d_6) δ 159.61, 157.67, 154.68, 154.25, 149.39, 144.63, 129.72, 125.77, 120.39, 113.15, 53.82 (2C), 18.39, 15.33; HRMS: $C_{14}H_{10}ClN_5S$ $[M+H]^+$ calc. mass 316.0418, found 316.0420.

Preparation of ***15e (VU6008462)*** *(Scheme 1, Table 4):*

Prepared in a similar manner as analog **15f** to afford title compound (4.3 mg, 32% yield). ^{1}H NMR (400 MHz, $CDCl_3$) δ 8.73 (s, 1H), 8.67 – 8.59 (m, 3H), 7.37 – 7.31 (m, 2H), 4.90 (t, *J* = 8.9 Hz, 2H), 4.50 (dd, *J* = 8.7, 5.9 Hz, 2H), 4.06 (tt, *J* = 8.7, 5.9 Hz, 1H), 3.18 (s, 3H). ^{13}C NMR (101 MHz, $CDCl_3$) δ 160.08, 157.98, 155.15, 154.76, 150.44 (2C), 150.09, 149.43, 145.38, 130.44, 126.63 (2C), 122.00, 113.98, 57.19, 34.27, 29.71, 15.71; HRMS: $C_{18}H_{14}ClN_5S$ $[M+H]^+$ calc. mass 368.0731, found 368.0732.

Preparation of ***15g (VU6056615)*** *(Scheme 1, Table 4):*

Prepared in a similar manner as analog **15f** to afford title compound (12 mg, 40% yield). ES-MS $[M+1]^+$: 290.8. ^{1}H NMR (400 MHz, $CDCl_3$) δ 8.78 (s, 1H), 8.64 (s, 1H), 5.34 (s, 1H), 3.19 (s, 3H), 3.07 (ttd, *J* = 6.9, 3.6, 2.2 Hz, 1H), 1.12 – 0.94 (m, 2H), 0.83 – 0.74 (m, 2H). ^{13}C NMR (101 MHz, $CDCl_3$) δ 159.96, 158.92, 155.44, 154.92, 149.56, 145.44, 130.56, 127.36, 114.71, 24.31, 15.84, 8.95 (2C); HRMS: $C_{13}H_{11}ClN_4S$ $[M+H]^+$ calc. mass 291.0466, found 291.0464.

Preparation of ***15h (VU6008460)*** *(Scheme 1, Table 4)*:

Prepared in a similar manner as analog **15f** to afford title compound (11 mg, 88% yield). ES-MS $[M+1]^+$: 333.0. ^{1}H NMR (400 MHz, $CDCl_3$) δ 8.69 (s, 1H), 8.63 (s, 1H), 4.91 (s, 4H), 4.62 (s, 4H), 3.16 (s, 3H). ^{13}C NMR (101 MHz, $CDCl_3$) δ 160.06, 157.67, 155.08, 154.73, 149.41, 145.36, 130.43, 126.64, 113.96, 80.80 (2C), 60.32 (2C), 39.39, 15.68; HRMS: $C_{15}H_{13}ClN_4OS$ $[M+H]^+$ calc. mass 333.0571, found 333.0573.

General Procedure for the Preparation of Analogs **22** (Scheme 2).

Synthesis of Intermediate ***16****:*

To a solution of cupric bromide (3.92 g, 18 mmol, 1.8 eq.) and *tert*-butyl nitrite (1.63 mL, 14 mmol, 1.4 eq.) in MeCN (49 mL) was slowly added intermediate **10a** (2.99 g, 9.8 mmol, 1.0 eq.) over 2 minutes and then stirred for 3 hours. The solution was then diluted with water (100 mL) and a precipitate was formed. The solid was collected by vacuum filtration, washed with water (3x), and dried in a vacuum oven overnight to yield title compound which was carried forward without further purification (2.99 g, 88% yield). LRMS: $C_{12}H_{11}BrClNO_2S$ $[M+H]^+$ calc. mass 347.9, found 348.2/350.2.

Synthesis of Intermediate ***18****:*

A mixture of ethyl intermediate **16** (1.46 g, 4.2 mmol, 1.0 eq.), vinylboronic acid pinacol ester (1.07 mL, 6.3 mmol, 1.5 eq.), Pd(dppf)Cl_2 (154 mg, 0.21 mmol, 0.05 eq), and Cs_2CO_3 (4.10 g, 13 mmol, 3.0 eq.) in THF (40 mL) and water (4 mL) was microwave irradiated at 130 °C for 35 minutes. After cooling to room

temperature, the reaction was diluted with water and extracted with EtOAc (3x). The organics were combined, washed with water (3x), dried ($MgSO_4$), filtered, and concentrated under reduced pressure. Purification via normal-phase column chromatography on silica gel (0-25% EtOAc/hexanes) provided title compound (1.24 g, 99% yield). ^{1}H NMR (400 MHz, $CDCl_3$) δ 7.16 (dd, *J* = 17.7, 11.4 Hz, 1H), 5.67 (dt, *J* = 11.3, 1.7 Hz, 1H), 5.37 (dt, *J* = 17.4, 1.7 Hz, 1H), 4.36 (q, *J* = 7.1 Hz, 2H), 2.74 (s, 3H), 2.73 (s, 3H), 1.39 (t, *J* = 7.1 Hz, 3H); LRMS: $C_{14}H_{14}ClNO_2S$ $[M+H]^+$ calc. mass 296.0, found 296.2.

*Synthesis of Intermediate **19**:*

To a solution of intermediate **18** (1.24 g, 4.2 mmol, 1.0 eq.) in DCM (20 mL) at -78 °C, was bubbled ozone for 45 minutes. After the ozone source was removed, dimethyl sulfide (2.17 mL, 29 mmol, 7.0 eq.) was added and the mixture was allowed to warm slowly to room temperature and stirred for 16 h. The solution was then washed sequentially with water (3x) and brine, then passed through a phase separator and concentrated *in vacuo*. Purification via normal-phase column chromatography on silica gel (0-25% EtOAc/hexanes) provided title compound (476 mg, 38%). ^{1}H NMR (400 MHz, $CDCl_3$) δ 10.81 (t, *J* = 1.1 Hz, 1H), 4.45 (q, *J* = 7.1 Hz, 2H), 2.76 (s, 3H), 2.61 (s, 3H), 1.44 (t, *J* = 7.1 Hz, 3H); LRMS: $C_{13}H_{12}ClNO_3S$ $[M+H]^+$ calc. mass 298.0, found 298.1.

*Synthesis of Intermediate **20**:*

To a solution of intermediate **19** (476 mg, 1.6 mmol, 1.0 eq.) in EtOH (20 mL) and IPA (20 mL) was added hydrazine (1.26 mL, 40= mmol, 25 eq.) and the mixture was heated to 110 °C overnight. After cooling to ambient temperature, the reaction mixture was added to water (10 mL) and a precipitate was observed. The solid was collected by vacuum filtration and washed with water to give title compound (364 mg, 86% yield). ^{1}H NMR (400 MHz, DMSO-d_6) δ 8.87 (d, *J* = 1.3 Hz, 1H), 2.97 (d, *J* = 1.4 Hz, 3H), 2.74 (s, 3H); LRMS: $C_{11}H_8ClN_3OS$ $[M+H]^+$ calc. mass 266.0, found 266.0.

*Synthesis of Intermediate **21**:*

A solution of intermediate **20** (329 mg, 1.2 mmol, 1.0 eq.) in $POCl_3$ (16.4 mL, 18 mmol, 14 eq.) was microwave irradiated for 20 minutes at 150 °C. After cooling to ambient temperature, the reaction mixture was diluted with DCM and cooled to 0 °C. The solution was slowly diluted with water, followed by the dropwise addition of a saturated aqueous $NaHCO_3$ solution. Next, small aliquots of powdered K_2CO_3 was added until the pH > 7 was obtained. The solution was extracted with DCM (3x) and the pooled organics were passed through a phase separator then concentrated to afford title compound (277 mg, 75% yield). ^{1}H NMR (400 MHz, $CDCl_3$) δ 9.89 (s, 1H), 3.06 (s, 3H), 2.85 (s, 3H); LRMS: $C_{11}H_7Cl_2N_3S$ $[M+H]^+$ calc. mass 284.0, found 284.0.

Preparation of ***22i (VU6008650)*** *(Scheme 1, Table 2):*

A solution of intermediate **21** (10 mg, 0.04 mmol, 1.0 eq), cyclopropylamine (25 µL, 0.43 mmol, 12 eq.), and *N,N*-diisopropylethylamine (25 µL, 0.14 mmol, 4.1 eq.) in NMP (1 mL) was heated to 200 °C for 20 minutes. After cooling to ambient temperature, the solution was filtered and purified by RP-HPLC (20 - 60% ACN/water/0.05% NH_4OH) to afford title compound as the free base. The solid was dissolved in DCM and to the solution was added a couple of drops of 4*N* HCl in dioxanes. The sample was concentrated to yield the title compound as an HCL salt (2.4 mg, 20% yield). ^{1}H NMR (400 MHz, DMSO-d_6) δ 9.45 (s, 1H), 7.52 (d, J = 2.9 Hz, 1H), 3.05 – 3.01 (m, 1H), 3.00 (s, 3H), 2.74 (s, 3H), 0.86-0.77 (m, 2H), 0.68 – 0.59 (m, 2H). ^{13}C NMR (101 MHz, DMSO-d_6) δ 158.71, 157.30, 155.37, 143.43, 139.16, 131.67, 129.61, 125.75, 124.67, 24.93, 24.46, 18.37, 7.38 (2C); HRMS: $C_{14}H_{13}ClN_4S$ $[M+H]^+$ calc. mass 305.0650, found 305.0623.

Preparation of ***22j (VU6008346)*** *(Scheme 1, Table 2):*

Prepared in a similar manner as analog **22i** to afford title compound (4.3 mg, 39% yield). ^{1}H NMR (400 MHz, DMSO-d_6) δ 9.38 (s, 1H), 8.02 (s, 1H), 4.66 (h, J = 7.9 Hz, 1H), 2.95 (s, 3H), 2.71 (s, 3H), 2.44 – 2.33 (m, 2H), 2.24 – 2.09 (m, 2H), 1.85 – 1.61 (m, 2H). ^{13}C NMR (101 MHz, DMSO-d_6) δ 158.35, 157.26, 152.44, 143.02, 137.44, 131.51, 129.36, 126.06, 125.11, 46.70, 29.99 (2C), 24.00, 17.83, 14.94; HRMS: $C_{15}H_{15}ClN_4S$ $[M+H]^+$ calc. mass 319.0779, found 319.0781.

Preparation of ***22k (VU6008649)*** *(Scheme 1, Table 2):*

Prepared in a similar manner as analog **22i** to afford title compound (4.6 mg, 34% yield). ^{1}H NMR (400 MHz, DMSO-d_6) δ 9.45 (s, 1H), 7.70 (d, J = 5.6 Hz, 1H), 4.53 (p, J = 6.5 Hz, 1H), 3.19 – 3.02 (m, 2H), 2.99 (s, 3H), 2.88 – 2.76 (m, 2H), 2.73 (s, 3H). ^{13}C NMR (101 MHz, DMSO-d_6) δ 158.64, 157.45, 153.59, 143.52, 139.43, 131.85, 129.71, 125.73, 125.11, 42.75 (t, J_{CF} = 21.6 Hz), 36.72 (dd, J_{CF} = 19.1, 6.1 Hz), 24.44, 18.37; HRMS: $C_{15}H_{13}ClF_2N_4S$ $[M+H]^+$ calc. mass 355.0590, found 355.0591.

Molecular Pharmacology Methods

Calcium Mobilization Assays.

To measure the functional activity of positive allosteric modulator (PAM) compounds in a cellular assay, human muscarinic receptor subtype 4 (M_4) or subtype 2 (M_2) was stably co-expressed with chimeric G_{qi5} protein in the Chinese hamster ovary (CHO) cells to evoke an increase in intracellular calcium to an EC_{20} concentration of acetylcholine (ACh) agonist. The stable M_4/G_{qi5}-CHO or M_2/G_{qi5}-CHO cells were

cultured in F12 medium containing 10% fetal bovine serum, 20 mM HEPES, 100 units/mL antibiotics/antimycotic, 0.5 mg/ml G418, and 0.2 mg/ml Hygromycin. All reagents used were from Life Technologies (Carlsbad, CA) unless otherwise noted.

Briefly, the day before the assay, stable M_4/G_{qi5}-CHO or M_2/G_{qi5}-CHO cells (15,000 cells/20 μL/well) were plated in black-walled, clear-bottomed, 384 well plates (Greiner Bio-One, Monroe, NC) in the culture medium without G418 and hygromycin, and then incubated overnight at 37 °C in the presence of 5% CO_2. The next day, calcium assay buffer (Hank's balanced salt solution (HBSS), 20 mM HEPES, 2.5 mM Probenecid, 4.16 mM sodium bicarbonate (Sigma-Aldrich, St. Louis, MO)) was prepared to dilute compounds, agonists, and Fluo-4-acetomethoxyester (Fluo-4-AM), fluorescent calcium indicator dye. Compounds were serially diluted 1:3 into 10-point concentration response curves in DMSO using the Bravo Liquid Handler (Agilent, Santa Clara, CA), transferred to a 384 well daughter plates using an Echo acoustic liquid handler (Beckman Coulter, Indianapolis, Indiana), and diluted in assay buffer to a 2X final concentration. The agonist plates were prepared using acetylcholine (ACh, Sigma-Aldrich, St. Louis, MO) concentrations for the EC_{20} and EC_{MAX} responses by diluting in assay buffer to a 5X final concentration. The 2X dye solution (2.3 μM) was prepared by mixing a 2.3 mM Fluo-4-AM stock in DMSO with 10% (w/v) pluronic acid F-127 in a 1:1 ratio in assay buffer. Using a microplate washer (BioTek, Winooski, VT), cells were washed with assay buffer 3 times to remove medium. After the final wash, 20 μL of assay buffer remained in the cell plates. Immediately, 20 μL of the 2X dye solution (final 1.15 μM) was added to each well of the cell plate using a Multidrop Combi dispenser (Thermo Fisher, Waltham, MA). After cells were incubated with the dye solutions for 45 min at 37 °C in the presence of 5% CO_2, the dye solutions were removed and replaced with assay buffer using a microplate washer, leaving 20 μL of assay buffer in the cell plate. The compound, agonist, and cell plates were placed inside the Functional Drug Screening System 7000 (FDSS7000, Hamamatsu, Japan) to measure the calcium flux. After establishment of a fluorescence baseline for 2-3 seconds (2-3 images at 1 Hz; excitation, 480 ± 20 nm; emission, 540 ± 30 nm), 20 μL (2X) of test compound or vehicle was added to the cells, and the response was measured. 140 seconds later, 10 μL (5X) of an EC_{20} concentration of ACh (Sigma-Aldrich, St. Louis, MO) or vehicle was added to the cells, and the response of the cells was measured. Approximately 125 seconds later, an $EC_{80\ or}$ EC_{MAX} concentration of ACh was added. Calcium fluorescence was recorded as fold over basal fluorescence and raw data were normalized to the maximal response to agonist. Calcium fluorescence was recorded as fold over basal fluorescence and raw data were normalized to the maximal response to ACh agonist. Potency (EC_{50}) and maximum response (% ACh Max) for compounds was determined using a four-parameter logistical equation using GraphPad Prism (La Jolla, CA) or the Dotmatics software platform (Woburn, MA):

$$y = bottom + \frac{top - bottom}{1 + 10^{(LogEC50 - A)Hillslope}}$$

where A is the molar concentration of the compound; *bottom* and *top* denote the lower and upper plateaus of the concentration-response curve; HillSlope is the Hill coefficient that describes the steepness of the curve; and EC_{50} is the molar concentration of compound required to generate a response halfway between the *top* and *bottom*.

DMPK Methods

Binding in plasma from rat and human.

Determination of compounds' fraction unbound (f_u) in plasma from rat and human was conducted *in vitro* via equilibrium dialysis using HTDialysis membrane plates. Dialysis membranes (four paired strips per HTD assay) were hydrated as described by the manufacturer and inserted into the HTD plate, which was assembled and prepared for sample addition by the dispensing of blank buffer (DPBS, 100 µL/well) into the 'top half' of each membrane-split well. Each compound was diluted into plasma from each species (5 µM final concentration), which was aliquoted in triplicate to the 'bottom half' of the prepared HTD plate wells. The HTD plate was sealed and incubated for 6 hours at 37 °C. Following incubation, each well (both top and bottom halves) were transferred (20 µL) to the corresponding wells of a 96-shallow-well (V-bottom) plate. The daughter plates were then matrix-matched (buffer side wells received equal volume of plasma, and plasma side wells received equal volume of buffer), and extraction solution (120 µL; acetonitrile containing 50 nM carbamazepine as IS) was added to all wells of both daughter plates to precipitate protein and extract test article. The plates were then sealed and centrifuged (3500 rcf) for 10 minutes at ambient temperature. Supernatant (60 µL) from each well of the daughter plates was then transferred to the corresponding wells of new daughter plates (96-shallow-well, V bottom) containing water (Milli-Q, 60 µL/well), and the plates were sealed in preparation for LC-MS/MS analysis (see below).

f_u was calculated as (analyte to IS MS peak area ratio from Trans-buffer side) / (analyte to IS MS peak area ratio from Cis-plasma side). Mean values for each species were calculated from 3 replicates.

Binding in brain homogenate from rat.

Determination of fraction unbound (f_u) in brain homogenate from rat was conducted using the same methodology and procedure than described for plasma protein binding assay with the following modifications: 1) a final compound concentration of 1 µM was used, 2) naïve rat brains were homogenized in DPBS (1:3 composition of brain: DPBS, w/w) using a Mini-Bead Beater™ machine in order to obtain brain homogenate.

The diluted fraction unbound (f_{u2}) in brain was calculated as (analyte to IS MS peak area ratio from Trans-buffer side) / (analyte to IS MS peak area ratio from Cis-brain homogenate side). Undiluted fraction unbound for the brain was calculated using the following equation:

$$f_u = \frac{1/4}{\left\{\left(\frac{1}{f_{u2}}\right) - 1\right\} + 1/4}$$

Mean values for each species were calculated from 3 replicates.

Intrinsic Clearance in Rat and Human Liver Microsomes

The *in vitro* intrinsic clearance (CL_{int}) was investigated in commercially obtained hepatic microsomes from rat and human donors using the substrate depletion (i.e., loss-of-parent vs. time, or $t_{1/2}$ method) approach with analyte detection via liquid chromatography-tandem mass spectrometry (LC-MS/MS). For each species, mean %parent remaining values at each time point were calculated from replicates raw data (analyte:IS peak area ratios) and used to determine *in vitro* $t_{1/2}$ and CL_{int}.

Experiments were carried out using a robot-assisted (TECAN model Evo 200). Compound was incubated (1 μM final concentration) in buffer (100 mM potassium phosphate pH 7.4 with 3 mM $MgCl_2$) containing hepatic microsomes (0.5 mg/mL final concentration) from multiple species, discretely, at 37 °C under constant orbital shaking. After 5 minutes (pre-incubation), reactions were initiated by addition of nicotinamide adenine dinucleotide phosphate (NADPH, 1 mM final concentration). At selected time intervals (0, 3, 7, 15, 25, and 45 minutes) post-addition of NADPH, aliquots (50 μL) were taken and placed into a 96-shallow-well plate containing ice cold acetonitrile (150 μL) with carbamazepine (IS, 50 nM). The plates were then centrifuged (3000 rcf at 4 °C) for 10 minutes. The supernatants were transferred to a new 96-shallow-well daughter plate and diluted (1:1 v/v) with water (Milli-Q filtered). The plates were then sealed in preparation for LC-MS/MS analysis (see below).

Raw LC-MS/MS peak area data generated from the assay samples were used to construct natural log-transformed %parent remaining vs. time plots (using t = 0 minute post-NADPH addition sample data as starting point set to 100%). *In vitro* compound half-life ($t_{1/2}$) values were obtained using the following equation:

$$t_{1/2} = \frac{Ln(2)}{k}$$

Where k is the slope from linear regression analysis of the natural log-transformed data (using means from all replicates at each time point). Resulting $t_{1/2}$ values were then used to calculate hepatic CL_{int} values according to the following equation and with the use of species-specific scale-up factors for liver weight (grams) per total body weight (kg):

$$CL_{int} = \frac{0.693}{in\ vitro\ t_{1/2}}\ x\ \frac{1\ mL\ incubation}{0.5\ mg\ microsomes}\ x\ \frac{45\ mg\ microsomes}{1\ gram\ liver}\ x\ \frac{^{a}\ gram\ liver}{kg\ body\ wt}$$

[a]Scale-up factors used are 45 (rat) and 20 (human).[1]

Predicted hepatic clearance (CL_{hep}) was calculated using the following equation:

$$CL_{hep} = \frac{Q_h * CL_{int}}{Q_h + CL_{int}}$$

Q_h represents hepatic blood flow (mL/min/kg): 21 for human, 70 for rat, and 90 for mouse.

<u>LC-MS/MS Analysis</u>

Prepared samples were injected (10 µL each) onto an AB Sciex Triple Quad 4500 mass spectrometer system with an Agilent 1260 Infinity II pump and autosampler. Mass spectrometer conditions are described in **Table S1.** Quantitation of compounds was performed via AB Sciex Multiquant software using the raw analyte:IS peak area ratios. The typical detection range was 0.5 ng/mL to ≥ 5,000 ng/mL utilizing a quadratic equation regression with 1/x2 weighting.

Correction for dilution of all brain samples (in extraction buffer and subsequently in blank plasma, as previously described) was performed post-quantitation. The corrections for dilution in extraction buffer employed correction factors specific to each brain weight (not shown).

Table S1. LC-MS/MS Conditions*

Injection volume	10 µL	
Mobile phase A	0.5% Formic Acid in Water	
Mobile phase B	0.5% Formic Acid in Acetonitrile	
Flowrate	0.5 mL/min	
Gradient	Time	% Mobile Phase B
	0.0	5
	0.2	5
	0.8	95
	1.5	95
	1.7	5
	2.7	Stop
Column	Fortis C18 (50 x 3.0 mm, 3 µm)	
Data collection and analysis software/version	Analyst v. 1.7.1	
Ionization mode	Positive Electrospray	
Curtain gas (psi)	40	
GS1 (psi)	40	
GS2 (psi)	40	
Capillary voltage (V)	5500	
Source TurboIonSpray® temp. (°C)	500	

References

1) Lin JH, Chiba M, Balani SK, Chen IW, Kwei GY, Vastag KJ, Nishime Ja. Species differences in the pharmacokinetics and metabolism of indinavir, a potent human immunodeficiency virus protease inhibitor. *Drug Metab. Dispos*. 1996;24:1111-1120.
